# The effectiveness of self-care interventions in working-age adults with type 2 diabetes mellitus: a systematic review and meta-analysis

**DOI:** 10.3389/fendo.2026.1941060

**Published:** 2026-09-11

**Authors:** Qingqing Zhu, Minqi Xia, Simin Zheng, Xianqi Gao, Xuefen Lan, Shunfei Lu

**Affiliations:** 1 Faculty of Nursing, College of Medicine, Lishui University, Lishui, China; 2 College of Medicine, Lishui University, Lishui, China

**Keywords:** meta-analysis, self-care, systematic review, type 2 diabetes, working-age adults

## Abstract

**Background:**

The prevalence of type 2 diabetes mellitus (T2DM) is rapidly increasing among working-age adults. This group faces unique challenges in sustaining effective self-care due to social and occupational demands. We conducted a systematic review and meta-analysis to evaluate the effectiveness of self-care interventions on glycemic control and self-care behaviors among working-age adults with T2DM.

**Methods:**

We searched nine databases (PubMed, Embase, CINAHL, Cochrane Library, Web of Science, CBM, CNKI, CQVIP, and Wan Fang) to identify randomized controlled trials (RCTs) published from January 2020 to April 2025. Eligible studies included 18-60-year-old adults with T2DM receiving self-care interventions. Review Manager 5.4 was employed to calculate pooled mean differences (MD) with 95% confidence intervals (CI). Risk of bias was assessed using the Cochrane RoB2 tool.

**Results:**

Forty-seven RCTs with 6,464 participants met the inclusion criteria. Compared to controls, self-care interventions significantly reduced HbA1c (MD −0.26, 95% CI −0.43 to −0.09), total cholesterol (MD −0.15, 95% CI −0.28 to −0.02), body mass index (MD −0.52, 95% CI −1.01 to −0.03), and increased high-density lipoproteins levels (MD 0.19, 95% CI 0.06 to 0.33). Improvements were also observed in self-care behaviors (SMD 0.74, 95% CI 0.62 to 0.86) and self-efficacy (SMD 1.14, 95% CI 0.51 to 1.76). No significant effects were found for fasting blood glucose or 2-hour plasma glucose. Substantial heterogeneity existed across studies.

**Conclusions:**

Self-care interventions improved glycemic control and related outcomes in working-age adults with T2DM, although high heterogeneity and variable study quality limited interpretation and generalizability. Future trials should adopt standardized reporting systems for interventions, robust design, and longer follow-up to better inform clinical practice and public health policy.

**Systematic Review Registration:**

https://www.crd.york.ac.uk/PROSPERO/view/CRD420251037017, identifier CRD420251037017.

## Background

Type 2 diabetes mellitus (T2DM) is a common chronic metabolic disorder with an increasing global prevalence. According to the International Diabetes Federation (IDF), approximately 589 million people were living with diabetes in 2024, a number projected to reach 853 million by 2045 (1, 2). The burden of diabetes is particularly notable among working-age adults (18–60 years), among whom the prevalence of T2DM is rapidly increasing (3). For the purpose of this review, the working-age population was operationally defined as adults aged 18–60 years (3, 4). This age range was adopted to focus the review on adults who commonly experience dual responsibilities of employment and family while often facing competing demands from work, study, and social roles (4–6). These pressures, together with irregular lifestyles, contribute to poor adherence to health-promoting behaviors and increase the risk of glycemic and metabolic complications (7).

Self-care has long been recognized as a cornerstone of diabetes management, and theories and models offer a structured framework to guide nursing interventions, enhance their effectiveness, and tailor them to individual needs (8). The middle-range theory of self-care of chronic illness conceptualizes self-care as three interrelated processes: maintenance (health-promoting behaviors), monitoring (ongoing symptoms and body surveillance), and management (response to changes in the health status) (9, 10). For individuals with T2DM, effective self-care behaviors, such as adherence to diet, exercise, blood glucose monitoring, and medication, are critical for preventing complications and maintaining quality of life (11).

Although previous systematic reviews and meta-analyses have examined the effectiveness of self-care interventions among adults with T2DM across broad age ranges, their findings may not adequately capture the distinctive needs and challenges of working-age individuals, who often experience competing occupational, family, and social demands. In addition, evidence derived from mixed-age populations may mask age-specific variations in intervention effectiveness, implementation strategies, and behavioral outcomes. Therefore, a focused synthesis targeting working-age adults is needed to generate more precise evidence for developing tailored diabetes self-care interventions in this population. To date, only one review evaluated the effectiveness of diabetes management interventions among working-age populations (12). However, the review only focused on interventions for improving physical activity. The effectiveness of self-care interventions on clinical and behavioral outcomes among working-age adults with T2DM remains uncertain. Furthermore, previous reviews have highlighted several gaps: limited attention to the unique challenges faced by younger adults, high emphasis on glucose monitoring and medication adherence, and less focus on physical activity, diet, and foot care, and inconsistent use of theoretical frameworks to guide interventions (13–15). Moreover, previous meta-analyses often included all age groups, limiting insights specific to working-age adults.

Given the growing burden of T2DM in this age group and the variability in intervention design and outcomes, a comprehensive synthesis is warranted. This systematic review and meta-analysis aimed to evaluate the effects of self-care interventions on glycemic and behavioral outcomes among working-age adults with T2DM. By focusing specifically on this population, our study sought to provide more targeted evidence to inform clinical practice, nursing care, and public health strategies.

## Methods

This systematic review and meta-analysis followed the *Preferred Reporting Items for Systematic Reviews and Meta-Analyses (PRISMA) 2020* guidelines (16). The protocol was prospectively registered in the International Prospective Register of Systematic Reviews (CRD420251037017).

### Search strategy

Nine databases were searched, including PubMed, Embase, CINAHL, the Cochrane Library, Web of Science, China Biology Medicine (CBM), China Knowledge Network (CNKI), CQVIP, and Wan Fang. The search covered English or Chinese studies published from January 2020 to April 2025. A systematic review confirmed the effectiveness of self-care intervention measures on the outcomes of chronic diseases (17). This study aimed to address the lack of a systematic review on the effectiveness of self-care intervention measures for patients with T2DM. Meanwhile, in line with previous systematic reviews on diabetes self-care interventions (18, 19), we only included studies published within the past 5 years (2020-2025) to ensure the representativeness and applicability of evidence. Gray literature was considered for inclusion, while studies with qualitative designs, descriptive cross-sectional studies, reviews, editorials, commentaries, letters, and experimental studies were excluded. Search terms combined controlled vocabulary and keywords for *type 2 diabetes*, *self-care/self-management*, *working-age adults*, and *randomized controlled trials*. The full electronic search strategy for PubMed is provided in **Supplementary File 1**. The search strategy was adapted for other databases accordingly.

### Inclusion and exclusion criteria

We included randomized controlled trials (RCTs), including cluster RCTs, measuring the effects of self-care interventions among adults with T2DM, with a primary focus on the working-age population (operationally defined as 18–60 years). Studies were eligible when the target population was not specifically restricted to older adults and was considered relevant to the working-age population. Eligible interventions were required to actively engage participants in self-care behaviors (e.g., monitoring, management, or maintenance) and could be delivered via any format (e.g., face-to-face, telephone, digital). Studies had to report at least one of the following outcomes: HbA1c (primary outcome), fasting blood glucose (FBG), 2-hour plasma glucose (2hPG), BMI, total cholesterol, high-density lipoproteins (HDL), self-care behaviors or self-efficacy. We excluded non-original studies (e.g., reviews, case reports), studies including only older adults, studies lacking a control group, and studies providing insufficient outcome data.

### Study selection and screening

Two reviewers (QQZ and XFL) independently screened titles and abstracts, followed by a full-text review for eligibility. Disagreements were resolved via consensus or consultations with a third reviewer (SFL). The selection process is summarized in a PRISMA 2020 flow diagram.

### Data extraction

Two reviewers (QQZ and XFL) independently extracted data using a standardized form. The following data were extracted: study characteristics (first author, year, country, setting, sample size, and participant demographics), intervention details (theory use, mode of delivery, components, duration, and behavior change techniques), comparator type, and outcomes (baseline and follow-up values and effect estimates). Discrepancies were resolved through discussion.

### Risk of bias assessment

**Figure 1:**
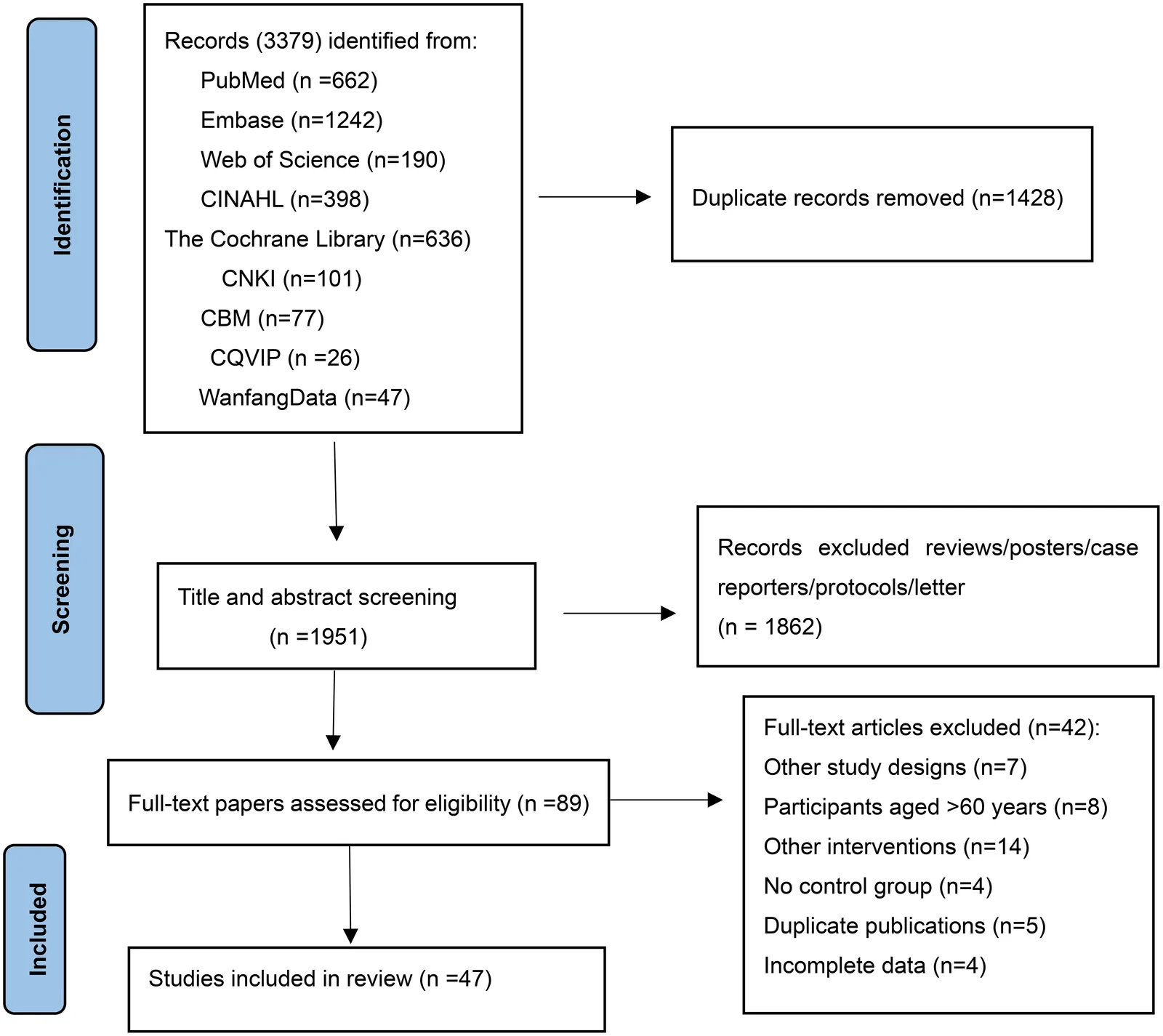
Flow diagram of study selection process.

Two reviewers (QQZ and XFL) independently assessed the methodological quality of the included studies using the Cochrane Risk of Bias 2 (RoB 2) tool (20). Each trial was evaluated across domains: randomization, allocation concealment, blinding of participants/personnel, blinding of outcome assessors, incomplete outcome data, selective reporting, and other sources of bias. The results were summarized graphically in **Figure 1**. The complete risk of bias ratings for included articles may be found in **Supplementary File 2**.

### Data synthesis and analysis

For continuous outcomes, mean differences (MD) with 95% confidence intervals (CI) were pooled using a random-effects model in Review Manager 5.4 (Cochrane Collaboration) (8). Since the indicators of self-care behaviors and self-efficacy were reported using different scales, standardized mean differences (SMD) were calculated to evaluate the intervention effects. Heterogeneity was assessed using the χ² test and I² statistic, with I² ≥50% indicating substantial heterogeneity (21, 22). Prediction intervals were calculated where appropriate. Subgroup analyses were pre-specified to explore the effect of (a) theoretical framework use and (b) length of intervention (≤3 months, 3–6 months, 6–12 months, and >12 months). Sensitivity analyses were conducted by excluding cluster RCTs and trials with a high risk of bias. Publication bias was assessed visually using funnel plots when ≥10 studies were available (23).

## Results

### Study selection process

In total, 3,379 records were retrieved across nine databases. After removing 1,428 duplicates, 1,951 titles and abstracts were screened. Among the 89 full-text articles assessed for eligibility, 42 were excluded for the following reasons: other study designs (n=7), participants aged >60 years (n=8), unrelated interventions (n=14), lack of a control group (n=4), duplicate publications (n=5), and incomplete data (n=4). Finally, 47 RCTs were included in this systematic review and meta-analysis (**Figure 1**).

### Characteristics of included studies

The 47 included studies (6,464 participants) were published between 2020 and 2025. Three used cluster randomization (24–26), while the remainder were individual RCTs. Among the 47 included RCTs, 41 studies (87.2%) were published in English and 6 studies (12.8%) were published in Chinese. Most were conducted in Asia (70%) (24–51). Other studies were conducted in Europe (19%) (52–60), North America (6%) (57, 61, 62), Oceania (2%) (63), and Africa (2%) (64). Study settings varied, including diabetes clinics, endocrinology departments, health centers, communities, and primary care. Sample sizes ranged from 40 (63) to 481 participants and varied across individual studies (26). For specific outcomes, the number of participants included in each meta-analysis differed due to variations in outcome reporting. The characteristics of the 47 included studies is provided in **Supplementary File 3**.

### Quality assessment of the included studies

**Figure 2:**
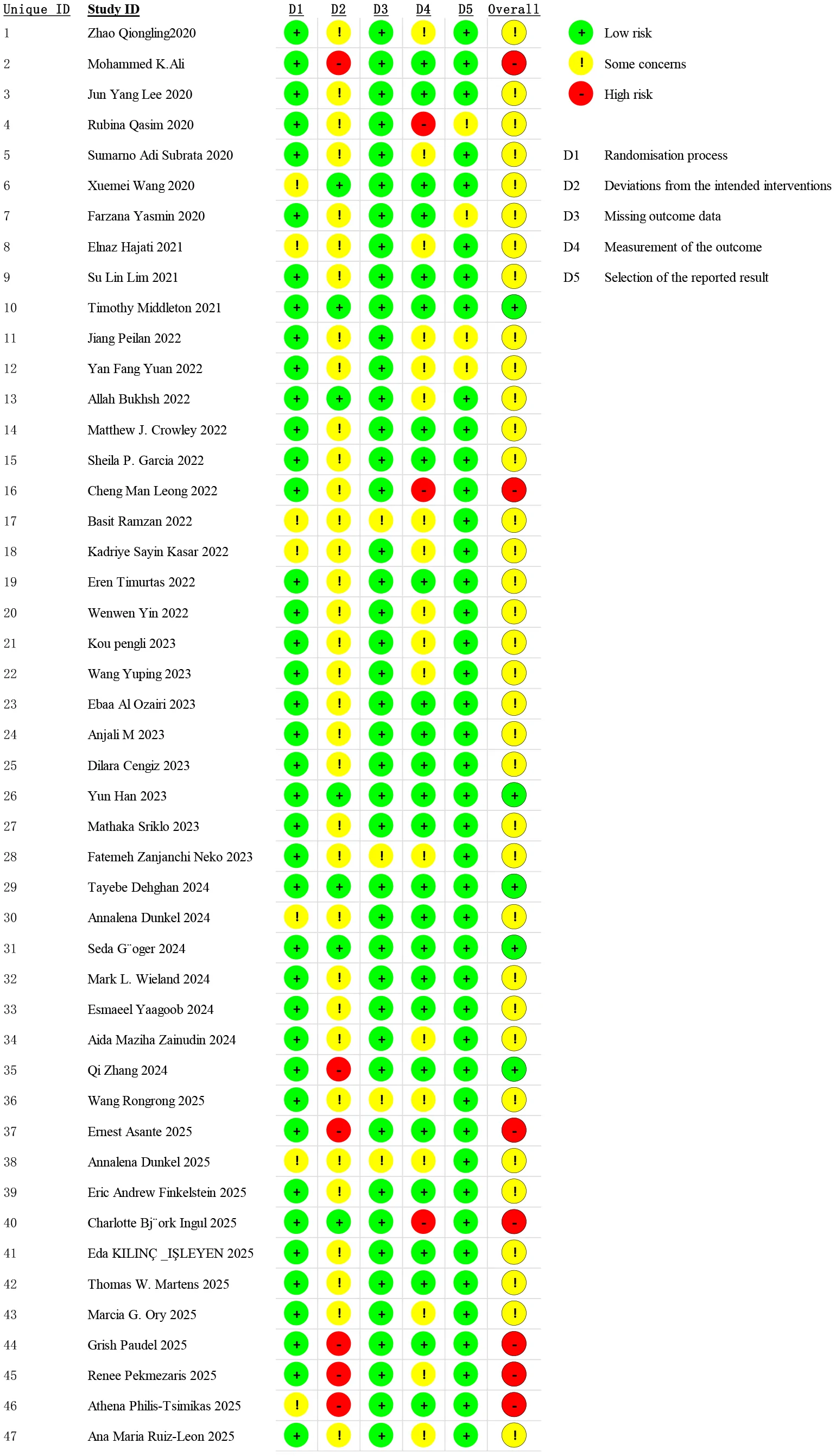
Risk of bias overview of RCTs.

As systematically evaluated using the Cochrane Risk of Bias 2 (RoB 2) tool (**Figure 2**), the 47 included RCTs demonstrated relatively robust methodological quality in several domains. For the randomization process, 40 studies (85.1%) were rated as having a low risk of bias, whereas 7 studies (14.9%) were judged as having some concerns, mainly due to insufficient reporting of random sequence generation or allocation concealment procedures. Regarding deviations from intended interventions, only 7 studies (14.9%) were considered at low risk of bias, while 34 studies (72.3%) had some concerns and 6 studies (12.8%) were rated as high risk. This was primarily attributed to the difficulty of implementing participant and personnel blinding in behavioral interventions, where knowledge of intervention allocation may influence participants’ adherence and behaviors. For missing outcome data, 43 studies (91.5%) were assessed as having a low risk of bias, indicating that most studies reported relatively complete follow-up data with acceptable attrition rates. Four studies (8.5%) were judged as having some concerns due to incomplete reporting of missing data or unclear handling of attrition. For outcome measurement, 26 studies (55.3%) were rated as low risk, 18 studies (38.3%) as having some concerns, and 3 studies (6.4%) as high risk. The main reasons included inadequate description of assessor blinding and the potential influence of subjective assessment methods, particularly for behavioral outcomes. Regarding selection of the reported results, 43 studies (91.5%) showed a low risk of bias, while 4 studies (8.5%) had some concerns because of insufficient information regarding prespecified outcomes or analysis plans. Overall, 5 studies (10.6%) were judged as having a low risk of bias, 35 studies (74.5%) raised some concerns, and 7 studies (14.9%) were considered at high risk of bias. The major sources of potential bias were related to deviations from intended interventions and insufficient blinding, which are common methodological challenges in behavioral intervention trials.

### Self-care intervention characteristics

Characteristics of the interventions and participants are described in **Table 1**. Nearly half of the trials (46.8%) were not explicitly informed by theory (28, 29, 32, 36, 37, 40–43, 49, 51, 52, 55, 57, 59, 61–63, 65–68). A majority of trials (65.9%) targeted all three components of self-care (maintenance, monitoring, and management) (25–27, 29–32, 34, 35, 38, 40–42, 44–49, 51–55, 59–62, 64, 65, 68). Various instructional methods were adopted, including lectures, group discussions, demonstrations, peer and family support, and distribution of educational materials. Group face-to-face (used in 31.9% of trials) and telephone (used in 44.7% of trials) were the two most common modes of delivery. Each intervention employed one or multiple behavioral change techniques from five different domains. Behavioral change techniques from two behavioral change domains were commonly employed: monitoring and recording of self-care behavior (in 85.1% of the included trials) and instruction on how to perform a behavior (in 91.5% of the included trials). The duration of interventions ranged from one month (31) to two years (56), with individual sessions lasting between 30 (27) and 120 min (67). The intervention sessions were held weekly (37), biweekly (26, 39), or monthly (53) in different trials, and follow-ups were conducted every month (61) or three months (24, 64).

**Table 1:** Characteristics of included randomized controlled trials (n = 47).

Number	First author (year)	Country/setting	Sample size IG/CG (n)	Age IG/CG(years)	Theory used	Mode of delivery	Main intervention components	Duration	Self-care components	Behavior change techniques applied	Comparator	Primary outcomes
1	Zhang Qi (2024) (25)	China/primary care	64/70	52.90 ± 6.93/50.92 ± 8.90	Nudge theory	face-to-face group	Dietary education, self-monitoring, feedback	3 mo	Maintenance, Monitoring and Management	Monitoring and recording of self-care behavior. instruction on behavior performance. credible sources	Routine education	HbA1c, BMI, diabetes distress; TC, FBG; TC; LDL-C; HDL-C; SBP; and DBP
2	Aida Maziha Zainudin(2024) (36)	Asia/endocrinology department	90/90	55.16 ± 7.93/57.08 ± 7.92	None	individual face to face	USM-IAM-based counselling	6 mo	Maintenance and Management	monitoring and recording of self-care behavior, instruction on behavior performance, social support, action planning,	Uasal care	HbA1c,FBS, IAQDM
3	Esmaeel Yaagoob(2024) (35)	Asia/diabetes clinics	40/40	41.55 ± 8.87/39.55 ± 6.99	Bandura’s theory of self-efficacy	skills training, telephone, self-monitoring, audio/visual online	WhatsApp-based DSMES	1.5 mo	Maintenance, Monitoring and Management	monitoring and recording of self-care behavior, instruction on behavior performance, social support, action planning, and credible sources	Usual care	DSMES-UK; DKT2
4	Mark L. Wieland(2024) (55)	Europe/health care centers	227/224	54.3 ± 9.3/54.5 ± 9.1	None	Video, telephone, DVD, flash drive, and web link	Digital Storytelling	3 mo	Maintenance, Monitoring and Management	instruction on behavior performance, social support, action planning	Usual care	BP, LDL, cholesterol, BMI, diabetes self-management behaviors.
5	Seda G¨oger(2024) (54)	Europe/diabetes clinics	45/47	None	planned behavior theory	face-to-face in groups; ppt; brochure; telephone	training to diabetes patients according to the theory	3 mo	Monitoring and Management; Maintenance	instruction on behavior performance, social support, action planning, and credible sources	Routine care	Diabetes Management Self-Efficacy Scale (DMSS), Patient empowerment scale (PES), HbA1C.
6	Annalena Dunkel(2024) (53)	Europe/endocrinology department	86/65	59.66 ± 6.24/58.80 ± 7.33	transtheoretical model	telephone, self-monitoring	lifestyle intervention, telemedically support, individual telephone coaching	24 mo	Monitoring and Management; Maintenance	monitoring and recording of self-care behavior, instruction on behavior performance, social support and credible sources	Routine care	HbA1c, BMI, physician contacts, costs for antidiabetics, physical activity and use of technology
7	Fatemeh Zanjanchi Neko(2023) (53)	Asia/health care centers	50/50	54.6 ± 0.6/55.7 ± 12.3	HAPA model	group skills training; telephone; face-to-face individual; online consultation, pamphlet, educational audio file	Educational intervention program	3 mo	Monitoring and Management; Maintenance	instruction on behavior performance, social support, action planning, and credible sources	Routine education	HbA1c,FBG, IPAQ-s
8	Mathaka Sriklo(2023) (33)	Asia/health-promoting hospitals	32/32	53.81 ± 5.18/52.66 ± 4.05	None	group face to face; a booklet	Transformative learning	3 mo	Management; Maintenance	instruction on behavior performance, social support, action planning, and credible sources	Usual care	HbA1C, the Self-Management Behaviors Scale, Health Literacy Scale
9	Dilara Cengiz(2023) (31)	Asia/endocrine department	24/27	52.9 ± 7.55/52.4 ± 6.72	Patient Health Engagement Model (PHE model)	face-to-face interview session, telephone consultation	reveal their awareness of the condition; follow up	1 mo	Monitoring and Management; Maintenance	monitoring and recording of self-care behavior, instruction on behavior performance, action planning	Usual care	HbA1C
10	Anjali M(2023) (30)	Asia/diabetes clinic	48/45	55.30 ± 10.5/51.45 ± 9.9	self-regulation theory, dual process theory, self-determination theory, and social learning theory.	App support. group face to face	DSME intervention	3 mo	Monitoring and Management; Maintenance	monitoring and recording of self-care behavior, instruction on behavior performance, social support, and credible sources	Routine education	HbA1C,BP,BMI,FBS, PPBS, DSMQ, DDS-17
11	Ebaa Al Ozairi(2023) (29)	Asia/Diabetes clinics	64/56	59.45 ± 8.55/60.99 ± 10.32	None	video call, video files, online exercise log, Online or phone text messages	pragmatic home‐based resistance exercise intervention	8 mo	Monitoring and Management; Maintenance	monitoring and recording of self-care behavior, instruction on behavior performance	Usual care	Grip strength, Total fat mass and total, arm, leg, and trunk lean mass, Liver fat
12	Yun Han(2023) (32)	Asia/endocrinology department	55/52	35.5 ± 9.2/38.2 ± 11.9	None	face-to-face individual, skill training group, online and on-site, self-monitoring, telephone	conventional course, face-to-face communication, activity training	12 mo	Monitoring and Management; Maintenance	monitoring and recording of self-care behavior, instruction on behavior performance, social support and credible sources	Conventional education	HbA1c,FPG,PPG, BMI, waist circumference, hip circumference, SBP and DBP
13	Wang Yuping(2023) (48)	Asia/endocrinology department	42/43	41.92 ± 8.31/42.45 ± 9.80	transtheoretical model	Group face-to-face, self-monitoring, exercise supervised pamphlet,	Exercise intervention and Motivational interviewing	3 mo	Monitoring and Management; Maintenance	monitoring and recording of self-care behavior, instruction on behavior performance, social support and credible sources	Usual care	HbA1c, Exercise Self-Efficacy Scale, ESES
14	Kou Pengli(2023) (50)	Asia/endocrinology department	36/36	55 ± 2.6/53 ± 0.5	Behavior change wheel	face-to-face individual, skill training group video files, PPT	Health education based on the BCW theory	3 mo	Management; Maintenance	instruction on behavior performance, social support, and credible sources	Routine education	HbA1c, Diabetes Self-Management Scale, Diabetes Quality of Life Questionnaire, Self-Perceived Burden Scale
15	Jiang Peilan (2022) (51)	Asia/endocrinology department	45/45	None	None	face-to-face individual, self-monitoring, Telephone follow-up, app support	Health education based on the 5A nursing model	6 mo	Monitoring and Management; Maintenance	monitoring and recording of self-care behavior, instruction on behavior performance, social support and credible sources	Routine education	HbA1c,2h-PG, FPG, SDSCA, DMQLS
16	Yuan Yanfang (2022) (47)	Asia/endocrinology department	40/40	None	the theory of happiness	Group face-to-face; skill training; online meeting; Diet Diary	Health education based on the theory of happiness	3 mo	Management; Maintenance; monitoring	monitoring and recording of self-care behavior, instruction on behavior performance, social support, action planning, and credible sources	Routine education	HbA1c, 2h-PG, FPG,BMI,CPSS, 2⁃DSCS
17	Wenwen Yin (2022)	Asia/endocrine department	60/60	47.17 ± 5.93/46.67 ± 6.67	None	telephone, telemedicine app	telemedicine management of diabetes	6 mo	Management; Monitoring,	monitoring and recording of self-care behavior, instruction on behavior performance, social support and credible sources	Usual care	HbA1c,FPG,PBG Self-Rating Depression Scale (SDS) score
18	Eren Timurtas (2022) (27)	Asia/diabetes clinics	30/30	51.8 ± 8.0/51.6 ± 7.8	Transtheoretical model	face-to-face group. skill training individual. self-monitoring, online platform	individually-tailored exercise regimen delivered through supervisor or one of the two technologies	3 mo	Management; Maintenance; monitoring	monitoring and recording of self-care behavior, instruction on behavior performance and credible sources	Usual care	HbA1c, Six Minute Walk Test (6MWT), exercise behavior, muscle function and physical capacity.
19	Kadriye Sayin Kasar (2022) (44)	Asia/endocrine department	40/40	52.90 ± 8.96/55.21 ± 8.72	IMB model	skills training individual, PPT, demonstration (role play) and discussion, text message (SMS),Telephone	telephone counseling	3 mo	Management; Maintenance; monitoring	monitoring and recording of self-care behavior, instruction on behavior performance, social support, action planning, and credible sources	Usual care	HbA1c,weight SBP, Type 2 Diabetes Self-Efficacy Scale score values;DSMQ,PDSMS
20	Basit Ramzan (2022) (43)	Asia/endocrine department	82/83	None	None	face-to-face group; Telephone	Group education and telephone contact	6 mo	Management; Maintenance	monitoring and recording of self-care behavior, instruction on behavior performance	Usual care	HbA1c, medication adherence, health-related quality of life, blood glucose, BP, and lipid profile
21	Cheng Man Leong (2022) (42)	Asia/endocrinology department	91/90	59.0 ± 11.4/58.1 ± 11.9	None	animated or filmed videos, animated or filmed videos. text message, voice, or video call	diabetes educational platform and LINE Oriented Video Education	3 mo	Management; Maintenance; monitoring	monitoring and recording of self-care behavior, instruction on behavior performance	Routine education	HbA1c, self-care activities.
22	Sheila P. Garcia(2022) (61)	North America/diabetes clinics	48/48	59 ± 9/60 ± 9	None	face-to-face individually meetings, Printed educational materials, electronic records.	Diabetes Self-Management Multidisciplinary Program (MP).	12 mo	Monitoring and Management; Maintenance	monitoring and recording of self-care behavior, instruction on behavior performance, social support, action planning, and credible sources	Routine education	HbA1c; Diabetes Quality of Life questionnaire, SDSCA, IPAQ, weight, BP, lipid profile
23	Matthew J. Crowley(2022) (52)	Europe/primary care	101/99	57.7 ± 8.3/57.8 ± 8.0	None	templated educational notes. telephone	Comprehensive Telehealth Intervention	12 mo	Monitoring and Management; Maintenance	monitoring and recording of self-care behavior, instruction on behavior performance, social support and credible sources	Usual care	HbA1c, diabetes distress, diabetes self-care, self-efficacy, BMI, and depression symptoms
24	Allah Bukhsh (2022) (41)	Asia/diabetes clinic	38/37	None	None	face-to-face individual, printed educational material and informatory brochures	Pharmacist-Led Intervention	6 mo	Monitoring and Management; Maintenance	monitoring and recording of self-care behavior, instruction on behavior performance, social support and credible sources	Usual care	HbA1c, disease knowledge and diabetes-related self-care practices
25	Timothy Middleton(2021) (63)	Oceania/Diabetes clinic Kadriye Sayin Kasar	20/20	33.0 ± 5.8/32.4 ± 4.4	None	Text message, support and reminder, study log, online platform	Enhanced SMS Text Message–Based Support and Reminder Program	12 mo	Management; Maintenance	instruction on behavior performance, social support	Usual care	100% attendance, metabolic indices, SMBG, psychosocial well-being.
26	Su Lin Lim(2021) (40)	Asia/Diabetes clinic	99/105	51.6 ± 9.4/50.8 ± 10.0	None	videos, via the app, self-monitoring	a smartphone-based lifestyle intervention	6 mo	Monitoring and Management; Maintenance	monitoring and recording of self-care behavior, instruction on behavior performance, social support, action planning and credible sources	Usual care	HbA1c, weight, FBG, BMI, total cholesterol, LDL, HDL, BP, creatinine levels, and dietary intake
27	Xuemei Wang (2020) (39)	Asia/endocrinology department	85/86	55.4 ± 9.7/54.7 ± 11.8	Trans-Theoretical Model (TTM)	Text message	SMS intervention	12 mo	Management; Maintenance	instruction on behavior performance	Usual care	HbA1c, changes in diet, physical activities, living habits, weight.
28	Sumarno Adi Subrata (2020) (38)	Asia/community	32/32	51 ± 5.10/51.20 ± 5.41	None	health education, skill training, and motivational interviewing	self- and family management support programs	3 mo	Monitoring and Management; Maintenance	monitoring and recording of self-care behavior, instruction on behavior performance, social support	Usual care	HbA1c, self-management, family supports, and wound size
29	Rubina Qasim (2020) (37)	Asia/Diabetic and Endocrinology campus	62/61	None	None	group face to face	Diabetic education using diabetes conversation maps	3 mo	Management; Maintenance	instruction on behavior performance	Routine education	HbA1c, DSME, DD
30	Jun Yang Lee(2020) (24)	Asia/primary care	120/120	56.1 ± 9.2/56.3 ± 8.6	The Health Belief Model (HBM)	self-monitoring, electronic logbook	home gluco-telemonitors and transmitted glucose data to a care team	18 mo	monitoring, Management	monitoring and recording of self-care behavior, instruction on behavior performance, social support and credible sources	Routine care	weight, FBG, BP, quality of life and physical activity
31	Qiongling Zhao (2020) (49)	Asia/endocrinology department	42/42	50.70 ± 8.68/50.78 ± 7.38	None	Telephone, face-to-face individual, WeChat APP, documents, pictures, audio and video to patients, online consultation	Motivational interviews conducted based on the WeChat platform	6 mo	Monitoring and Management; Maintenance	monitoring and recording of self-care behavior, instruction on behavior performance, social support and credible sources	Usual care	2h-PG, FPG, Diabetes Self-Management Behaviors, DD.
32	Elnaz Hajati (2021) (67)	Asia/endocrinology department	20/20	52.15 ± 3.20/53.55 ± 3.19	acceptance and commitment theory	Group face-to-face	Acceptance-based Emotion Regulation Group Therapy	6 mo	Management; Maintenance	monitoring and recording of self-care behavior, instruction on behavior performance, social support	Usual care	HbA1c, Diabetes Self-care Summary Questionnaire, Diabetes Dependent Quality of Life Scale, DSME
33	Mohammed K. Ali(2020) (65)	Asia/Diabetes clinic	196/208	52.1 ± 8.2/53.3 ± 8.9	None	Telephone, face-to-face individual, blood glucose logbook	collaborative care intervention	12 mo	Monitoring and Management; Maintenance	monitoring and recording of self-care behavior, instruction on behavior performance, social support and credible sources	Routine care	HbA1c, a 50% reduction in SCL-20, SBP, LDL
34	Farzana Yasmin (2020) (66)	Asia/endocrinology department	160/160	56.0 ± 40.7/52.0 ± 33.3	None	voice call, mobile phone	The m-health intervention	None	Management; Maintenance	instruction on behavior performance, social support and credible sources	Usual care	2h-PG, FPG
35	Rongrong Wng (2025) (68)	Asia/endocrinology department	48/48	48.29 ± 4.33/48.36 ± 4.17	Three-in-One Care Management Model	Telephone follow up, APP support,	Health education based on the Three-One Care Management Model	3 mo	Monitoring and Management; Maintenance	monitoring and recording of self-care behavior, instruction on behavior performance, social support and credible sources	Routine care	2h-PG, FPG, SDSCA, DMQLS
36	Ernest Asante(2025) (34)	Africa/Diabetes clinic	49/49	58.71 ± 11.04/57.76 ± 10.96	The transtheoretical model	telephone calls. Self-monitoring tools, booklet and audiovisual educational resources, educational materials, counseling, and support groups.	Nurse-led Mobile Phone Intervention	3 mo	Monitoring and Management; Maintenance	monitoring and recording of self-care behavior, instruction on behavior performance, social support and credible sources	Usual care	HbA1c, SDSCA
37	Annalena Dunkel(2025) (56)	Europe/primary care	86/65	59.66 ± 6.24/58.80 ± 7.33	The transtheoretical model	Self-monitoring tools, telephone, provided devices	The initiative diabetes Program	24 mo	Monitoring	monitoring and recording of self-care behavior, credible resources	Routine care	HbA1c, Self-Efficacy for Diabetes Scale (SED), Health-related quality of life((SF-12)
38	Eric Andrew Finkelstein(2025) (45)	Asia/Diabetes clinic	105/110	56.1 ± 8.38/54.9 ± 10.05	behavioral economic theory	mobile apps, self-monitoring tools	diabetes management program (DMP)	24 mo	Monitoring and Management; Maintenance	monitoring and recording of self-care behavior, instruction on behavior performance	Routine care	HbA1c, insulin treatment initiated
39	Charlotte Bj¨ork Ingul(2025) (46)	Europe/Diabetes clinic	57/53	58.4 ± 9.7/61.6 ± 8.8	None	face to face group, face to face individual, telephone, self-monitoring tools, information-materials and methods, online video meetings	Choice of lifestyle interventions	6 mo	Management; Maintenance	instruction on behavior performance, social support and credible sources	Routine education	HbA1c, waist circumference, 6minute walk test (6MWT), weight, cholesterol, LDL, high sensitivity C-reactive protein (hs-CRP), C-peptide, and Personal Activity Intelligence (PAI)
40	Thomas W. Martens(2025) (62)	North America/Diabetes clinic	41/31	55 ± 12/57 ± 13	None	face to face individual, skill training, self-monitoring tools,	CGM and food logging application	6 mo	Monitoring and Management; Maintenance	monitoring and recording of self-care behavior, action planning	Routine care	HbA1c, weight, time above range >180 mg/dL
41	Marcia G. Ory(2025) (57)	North America/community	60/61	50.3 ± 11.1/51.7 ± 11.9	None	face to face individual, registered dietician, mobile application, self-monitoring tools	self-paced virtual diabetes self-management interventions	6 mo	Monitoring and Management	monitoring and recording of self-care behavior, instruction on behavior performance, social support	Routine education	HbA1c
42	Grish Paudel(2025) (26)	Asia/community	238/243	None	RE-AIM (Reach, Effectiveness, Adoption, Implementation, Maintenance) framework	face to face group, reminder phone call and text messages, household visit, pictorial handout pictorial book	community-based lifestyle intervention	6 mo	Management and Monitoring, Maintenance	monitoring and recording of self-care behavior, instruction on behavior performance,social support, action planning	Usual care	self-care behaviors (adhering to a healthy diet, physical activity, medication adherence, glucose monitoring and foot care)
43	Renee Pekmezaris (2025) (58)	Europe/primary care	120/120	55.4 ± 41.5/54.8 ± 49.6	None	compliant tablet and Bluetooth, video, telehealth visits,diabetes educational videos	diabetes telehealth management (DTM)	6 mo	Monitoring, Maintenance	monitoring and recording of self-care behavior, instruction on behavior performance, social support, action planning, credible sources	Routine care	HbA1c
44	Athena Philis-Tsimikas(2025) (59)	Europe/cc	107/106	51.22 ± 10.7/53.21 ± 10.26	None	text messages, blood glucose monitor,phone calls	DD-Me-Auto(algorithm-driven text-based personalized feedback)	12 mo	Management and Monitoring, Maintenance	monitoring and recording of self-care behavior, instruction on behavior performance, social support, action planning,	Usual care	HbA1c, SBP,LDL
45	Ana Maria Ruiz-Leon(2025) (60)	Europe/primary care	61/62	56.0 ± 10.8/60.5 ± 8.11	the McClelland Iceberg model, the Behavioral Change Wheel,	App, email, face to face group, self-monitoring tools	12-week intervention with the Greenhabit behavioral treatment mHealth app	3 mo	Management and Monitoring, Maintenance	monitoring and recording of self-care behavior, instruction on behavior performance, social support, action planning, credible resources.	Routine care	HbA1c,FBG, BP, lipid profile, weight, adiposity, dietary habits, stress relief, and psychological factor
46	Tayebe Dehghan(2024) (82)	Asia/primary care	22/22	50·9 ± 6·77/52.89 ± 3·56	extended parallel process model (EPPM)	text messages, telephone calls, video clips, info graphs, face-to-face meetings	related intervention instructions for receiving the predefined messages	3 mo	Management and Monitoring, Maintenance	monitoring and recording of self-care behavior, instruction on behavior performance, social support, action planning, credible resources.	Routine care	FBS, HbA1C, 2h-PG, HDL and LDL, total cholesterol, TAG
47	Eda KILINÇ İŞLEYEN (2025) (46)	Asia/Diabetes clinic	30/30	54.0 ± 6.85/52.23 ± 5.58	IMB	education booklet, Computers, monitors, PowerPoint presentation, App video call.	IMB model-based - Diabetes Education - Motivational interview	3 mo	Management and Monitoring, Maintenance	monitoring and recording of self-care behavior, instruction on behavior performance, social support, action planning, credible resources.	Usual care	HbA1c and BMI. Diabetes Knowledge Scale (DKS), Diabetes Health Belief Model Scale (DHBMS)

HbA1c, glycated hemoglobin; 2h-PG, 2hour plasma glucose; BMI, body mass index; FBG, fasting blood glucose; HDL, high-density lipoprotein; LDL , Low-density lipoprotein BP, blood pressure; DSME, diabetes self-management education; IMB, information-motivation-behavioral skills model; SDSCA -Summary of Diabetes Self-Care Activities, PDSMS -Perceived Diabetes Self-Management Scale; DD -Diabetes Distress, mo – month(s).

### Narrative synthesis of outcomes

The primary outcome was HbA1c level, while secondary outcomes included fasting blood glucose (FBG), 2-hour plasma glucose (2hPG), body mass index (BMI), total cholesterol, high-density lipoproteins (HDL), self-care behaviors, and self-efficacy scores. Thirty-six studies reported significant decreases in HbA1c levels across varying follow-up periods (from immediately after the intervention to two years after the intervention). Self-care behaviors showed robust enhancement in thirteen studies, though measurement tools varied and could account for some differences in the reported effects. Five studies (42, 61, 64, 67, 68) used the validated SDSCA scale (69). Seven studies (30, 38, 44, 46, 49, 52, 68) employed diabetes self-management questionnaire (DSMQ), four studies (31, 35, 37, 54) used diabetes management self-efficacy scale (DMSES) (70), and two studies (44, 56) utilized established questionnaires, such as the Stanford education research center’s self-efficacy for diabetes scale (SED) (71) and the perceived diabetes self-management scale (PDSMS) (72). In 32 studies, improvements in HbA1c levels were linked to enhanced self-care behaviors, indicating that behavioral factors may play a key role in achieving better glycemic control. However, four studies (26, 29, 61, 63) showed that behavioral interventions may not significantly affect metabolic control or self-care behaviors.

Eight studies documented concurrent reductions in 2hPG and FPG (28, 30, 32, 39, 47, 49–51, 68). Metabolic outcomes exhibited variability, eight studies reporting BMI outcomes, which demonstrated statistically significant reductions (p<0.05). Seven out of the nine studies reporting HDL demonstrated clinically meaningful reductions. This pattern indicates that the intervention approach demonstrated a high degree of consistency in controlling glucose-related indicators, while its effects on other cardiometabolic risk factors showed considerable variability.

Differences in study design, such as different lengths of intervention and the theory used, may explain the discrepancies in outcomes. The intervention duration varied substantially among the included studies, ranging from 4 weeks (31) to 104 weeks (56). Most interventions lasted 3 months (n = 17), followed by 6 months (n = 10), 12 months (n = 7), and 24 months (n = 3). Explicit theoretical guidance revealed that heterogeneity was evident. With respect to the conceptual framework used to develop the interventions, four studies (27, 33, 39, 53) used a transtheoretical model, two studies (44, 46) employed an information-motivation-behavioral skills model, and three studies (30, 35, 38) adopted a self-management model. Three studies (48, 50, 60) used the behavioral change wheel model, whereas twenty-one articles did not report a theoretical or conceptual framework.

### Results of meta-analysis

#### Effect on HbA1c

**Figure 3:**
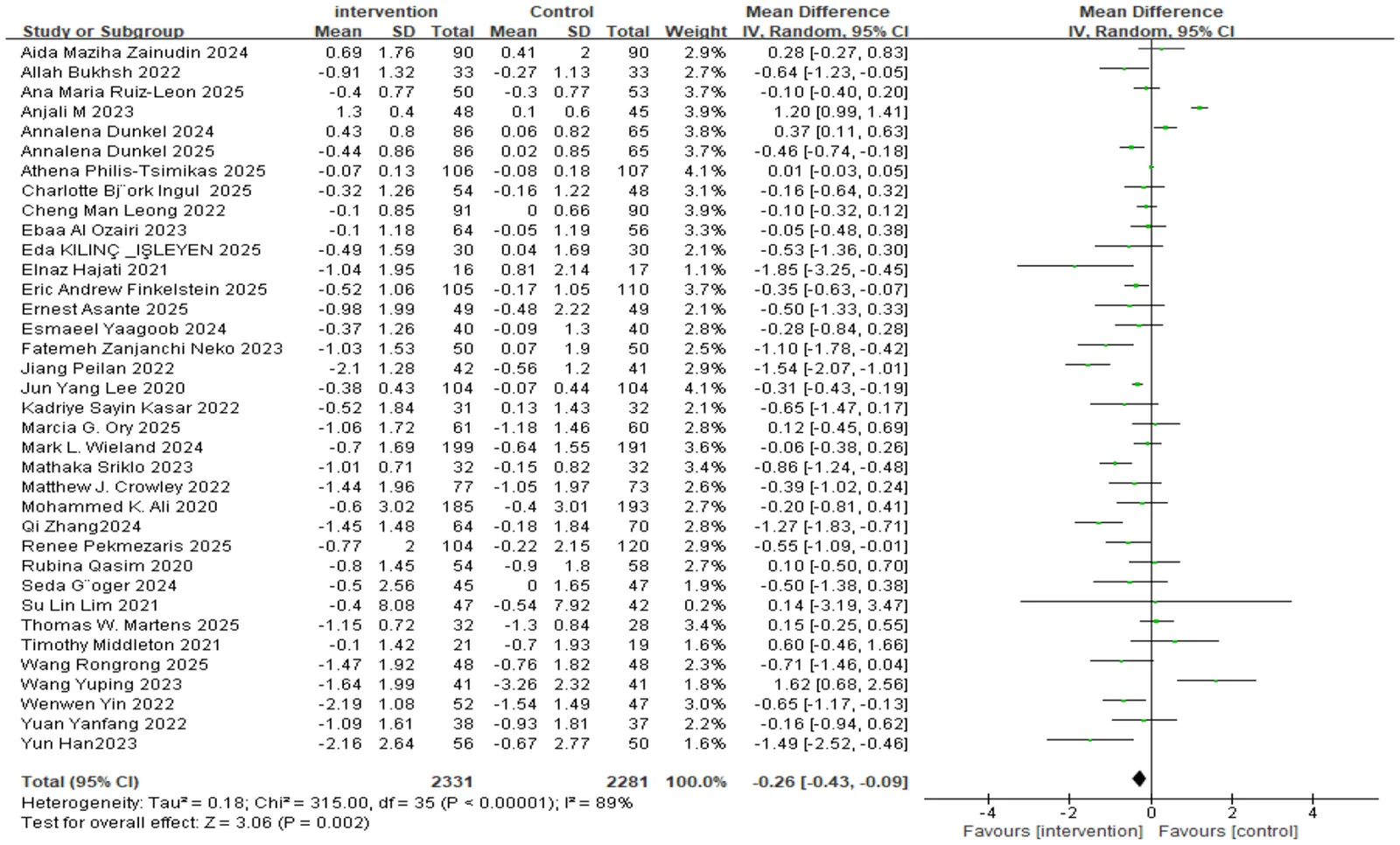
Forest plot for HbA1c.

**Figure 4:**
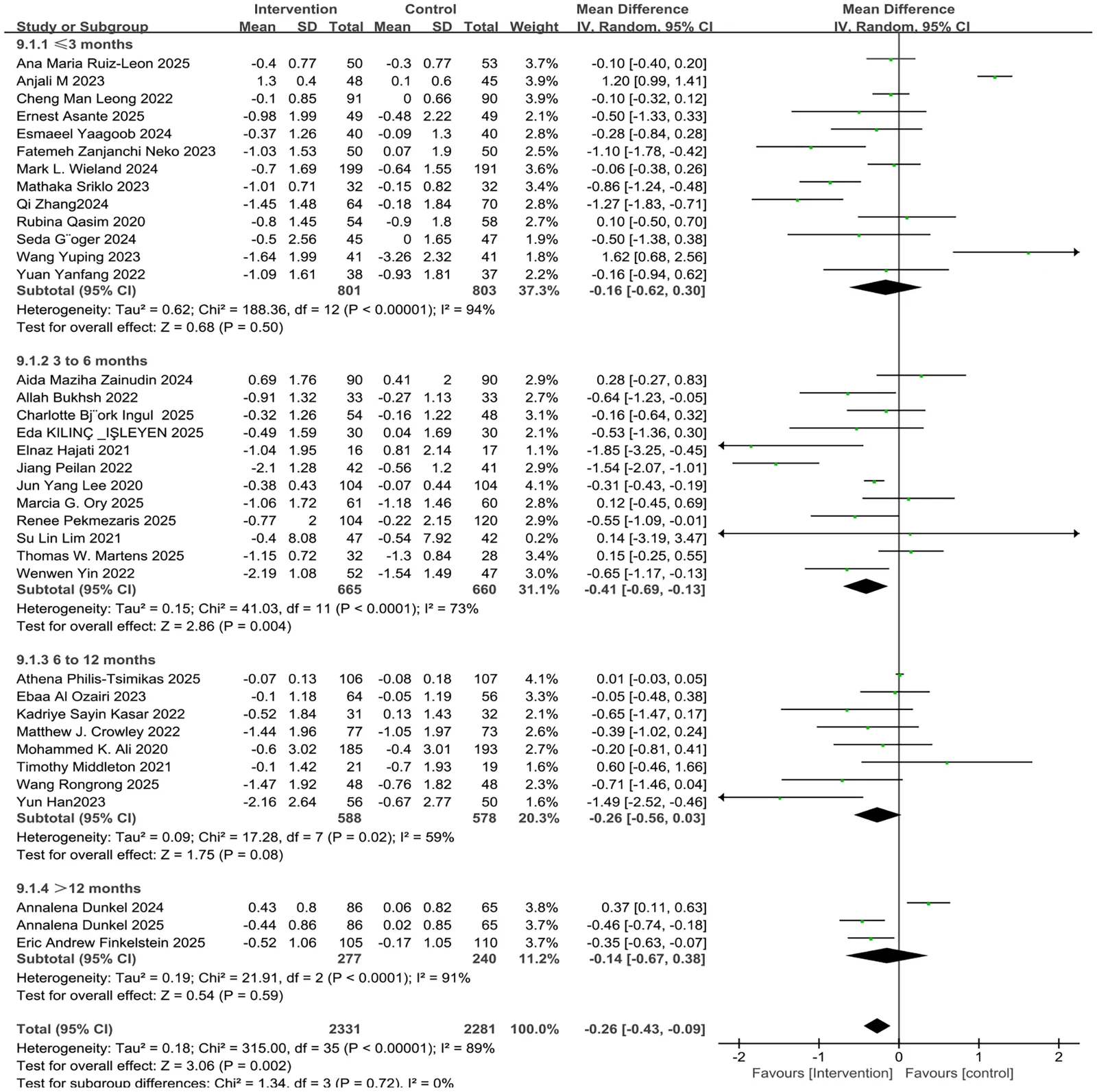
Sub-Analyses for HbA1c based on the length of intervention.

**Figure 5:**
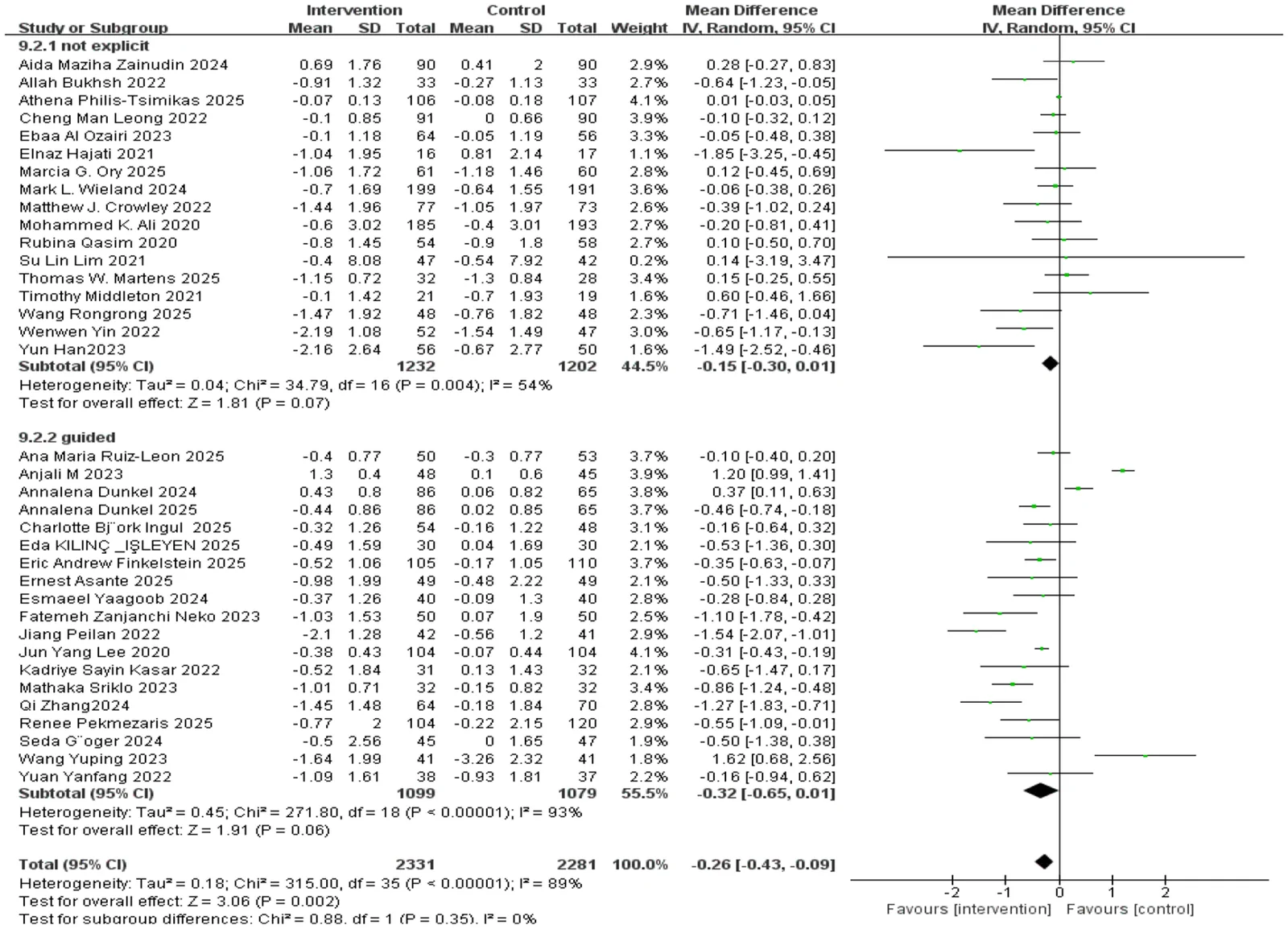
Sub-analyses for HbA1c based on the theory.

**Figure 6:**
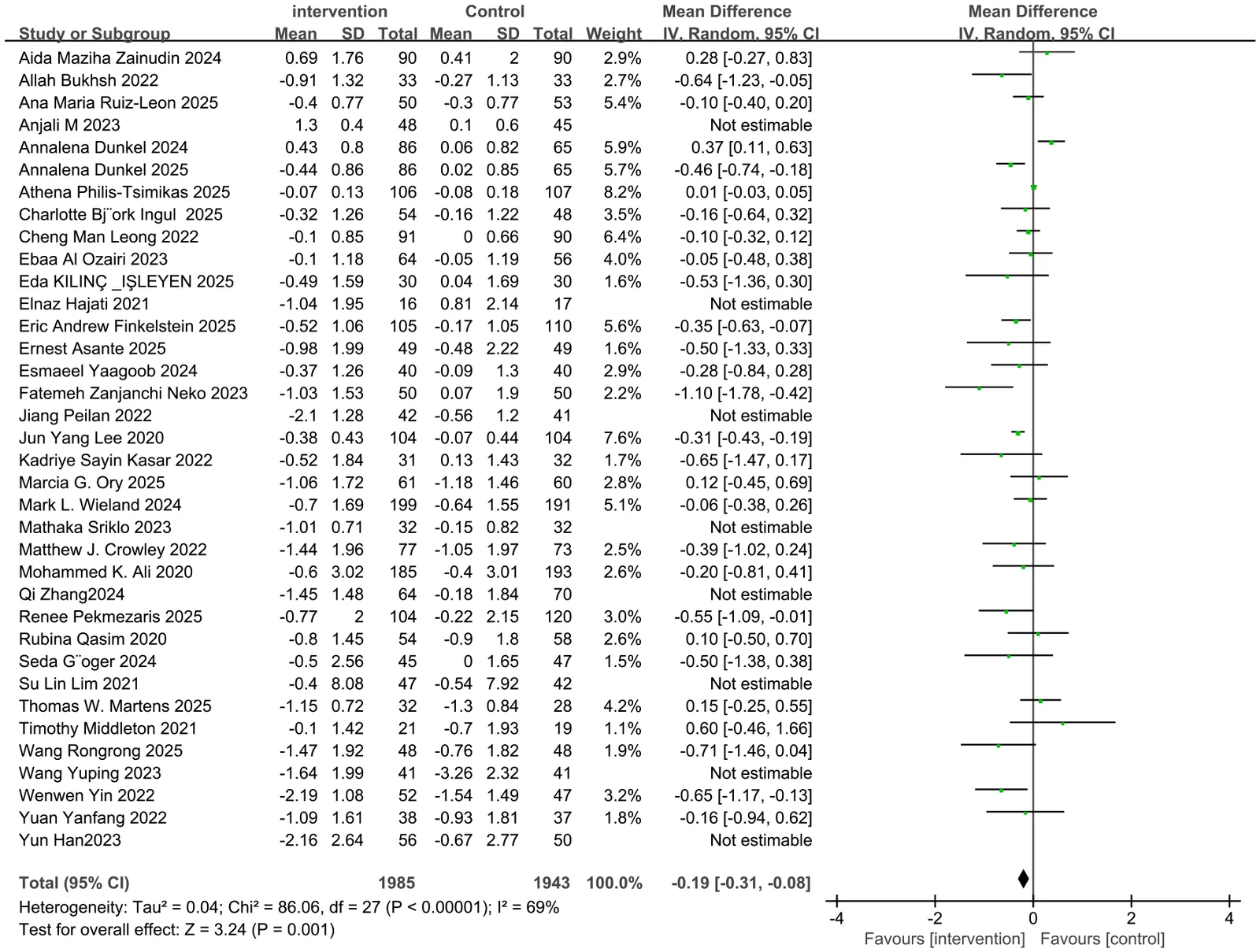
Results of sensitivity analysis.

Thirty-six studies assessed HbA1c, with a total of 4612 participants (2331 in the intervention group, 2281 in control group). Meta-analysis showed that the intervention group showed significantly lower HbA1c (MD= −0.26; 95% CI: −0.43 to −0.09; p =0.002), with substantial heterogeneity (I^2^ = 89%). Thus, an explorative explanation of the heterogeneity was found to be necessary (**Figure 3**). Thus, we conducted sub-analyses based on the length of intervention. The included studies were categorized into four groups:≤3 months(n =13), 3–6 months n=12), 6–12 months (n=8), and >12 months (n=3). For 3–6 months, the analysis (n =12 studies) indicated a significant decrease in HbA1c (MD = −0.41; 95% CI: −0.69 to −0.13; p=0.004) with high heterogeneity (I^2^ = 73%). As for 6–12 months, the change in HbA1c (n = 8 studies) was smaller (MD = −0.26; 95% CI: −0.56 to 0.03; p=0.08), with moderate heterogeneity (I^2^ = 59%). While no statistically significant differences were observed in the other duration categories (**Figure 4**). We also conducted sub-analysis to determine the effect of the theory based on the authors’ descriptions of their interventions, which showed significant differences (MD = -0.26, 95% CI: -0.43 to -0.09, P = 0.002) (**Figure 5**). To explore the sources of heterogeneity, sensitivity analysis identified Anjali (30), Hajati (67), Jiang (51), Mathaka (33), Zhang (25), Lim (40), Wang (48), Han (32) as the primary contributors, whose exclusion reduced I^2^ to 69% (MD = −0.19; 95% CI: −0.31 to −0.08) (**Figure 6**), possibly because the difference in HbA1c levels before and after the intervention was significant.

#### Effects on secondary outcomes

In addition to HbA1c, we measured other outcome variables, such as FBG, 2hPG, BMI, total cholesterol, HDL, self-care behaviors, and self-efficacy, as indicators associated with self-care. The intervention significantly increased self-care behaviors (13 studies) (SMD = 0.74; 95% CI: 0.62 to 0.86) and self-efficacy (8 studies) (SMD = 1.14; 95% CI: 0.51 to 1.76). Additionally, the intervention significantly decreased total cholesterol (MD =−0.15; 95% CI: −0.28 to−0.02) and BMI (MD = −0.52; 95% CI: −1.01 to −0.03; p=0.04) and increased HDL (MD = 0.19; 95% CI: 0.06 to 0.33). However, the meta-analysis revealed no significant improvement in FBG (MD = −0.61; 95% CI: −1.29 to 0.08; p=0.08) and 2hPG (MD = −0.89; 95% CI: −2.00 to 0.23; p=0.12).

### Publication bias

**Figure 7:**
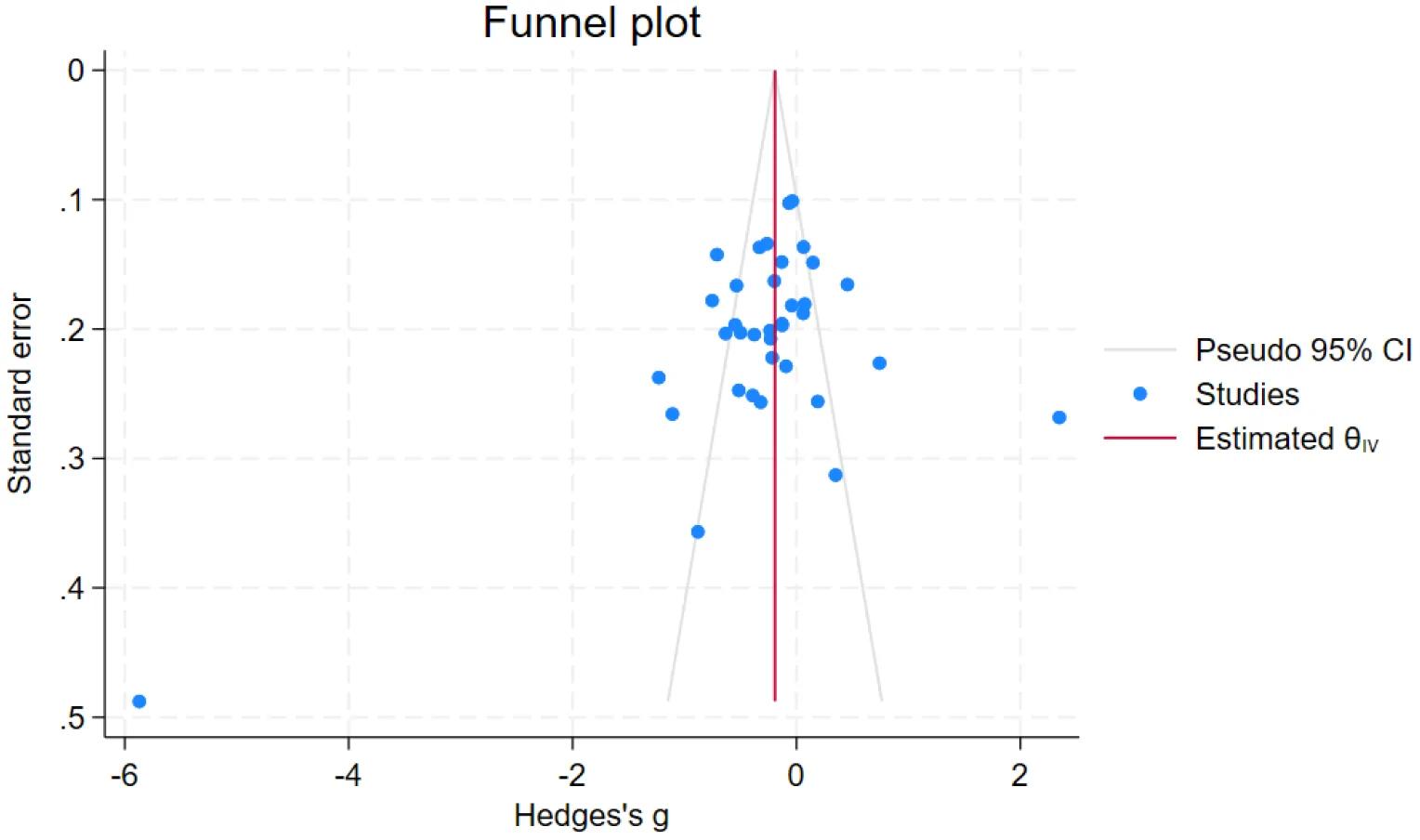
Funnel plot of standard error (SE) by mean difference (MD) when assessing the effect of self-care intervention on HbA1c.

As a measure of publication bias, funnel plots were drawn when there were 10 or more trials for any outcome (18). Egger’s regression test indicated significant funnel plot asymmetry for the HbA1c outcome (P = 0.0004), suggesting the presence of potential publication bias (**Figure 7**).

## Discussion

This systematic review and meta-analysis synthesized evidence from 47 RCTs with 6,464 working-age adults with T2DM. Self-care interventions were associated with significant improvements in HbA1c, BMI, total cholesterol, HDL, self-care behaviors and self-efficacy, although the effects on FBG and 2hPG were not significant. Subgroup analyses indicated that intervention duration and theory-guided approaches may affect the efficacy of self-care interventions, though heterogeneity remained high.

The middle-range theory of self-care, as proposed by Riegel (9), offers a structured framework that includes self-care maintenance, self-care monitoring, and self-care management. This theory maintains health with health-promoting practices within the context of the management needed for a chronic disease (73). The contents of the diabetes self-care intervention were a combination of two or more components, such as maintenance plus management, monitoring plus management, and maintenance monitoring plus management. The comprehensive intervention nature of diabetes treatment measures reflects the complexity of the management process (74). Most interventions included in this review did not employ a theoretical framework in their design. Among those employing a theoretical framework, the transtheoretical model and the behavior change wheel were the most frequently reported. This observation is consistent with earlier reviews, highlighting the limited application of theory in self-care interventions (75, 76). Doshmangir et al. reported that lifestyle interventions grounded in theories or models can significantly improve HbA1c in patients with T2DM (77). Similarly, Ayling et al. reported that interventions explicitly guided by theory may achieve greater efficacy than those lacking such a foundation (78). These findings highlight the potential value of the theoretical framework as a tool for promoting behavioral changes in this population (79, 80). Our analysis also indicated that there are significant differences in outcomes between theory-based intervention measures and non-theoretical intervention measures. This further emphasizes the need for systematically integrating theoretical guidance in future RCTs.

Self-care is an extremely challenging process. Several factors affect self-care, including experience, skill, motivation, culture, confidence, habits, function, cognition, support from others, and access to care (9). A study found self-care to be effective by increasing information, health beliefs, self‐efficacy, and self‐management, resulting in decreased HbA1c and BMI in adults with T2DM (46). Many self-care behaviors are triggered by motivators. A study explored the effect of motivational interviewing in exercise intervention for middle-aged and young patients with T2DM (48). Sometimes self-care advice may be inconsistent with cultural beliefs. A study took cultural factors into account, suggesting that culturally congruent diabetes telemonitoring may be effective for this underserved population (58). The support from others is also a crucial motivating factor (26, 65). A scoping review revealed that most interventions delivered by multidisciplinary teams were effective and improved HbA1c and other clinical outcomes, such as social support (81). Habits, function, and cognitive factors identified in previous studies were applied less frequently.

All the studies included in this meta-analysis used intervention programs targeting specific groups and provided practical guidance for future studies (82). Most studies employed multiple modes of delivery, with group face-to-face teaching and regular telephone follow-up being the most common modes of delivery. Group face-to-face teaching through diverse formats, such as health lectures, discussions, workshops, and interactive Q&A sessions, can help achieve better outcomes. This approach promotes peer collaborative learning and interactive communication (25, 32). It effectively enhanced the participants’ knowledge reserves, promoted the exchange of experiences, and shaped a positive perception and attitude toward disease management (27). Other supportive interventions, such as regular telephone follow-ups, text messages, and outpatient counselling, provided the necessary social support, thereby enhancing the compliance of the participants (42, 65). Furthermore, the use of tools, such as printed educational materials, self-monitoring tools, and diet diaries, enables participants to acquire the necessary self-management skills (32, 44, 54).

This study confirmed the findings of previous systematic reviews (19, 83, 84). It adopted a new theory to enhance the insights of this study, strengthening self-care behaviors, improving its key variables, and enhancing the efficacy of self-care interventions in reducing HbA1c levels in patients with T2DM. Moreover, our findings suggest that self-care interventions may improve both clinical and behavioral outcomes among working-age adults with T2DM, highlighting the importance of addressing behavioral determinants beyond glycemic control alone. This finding indicates sustained benefits and progressive improvements over time. This is in line with previous studies that have shown that interventions with longer durations are associated with better clinical outcomes (85). Heterogeneity was notably high after 3 months of follow-up (I^2^ = 94%), the variability in intervention effects across follow-up periods highlights the influence of contextual and implementation-related factors. Differences in participants’ baseline characteristics, adherence levels, intervention intensity, delivery frequency, and the type of intervention providers may contribute to variations in outcomes. For example, interventions delivered by nurses or multidisciplinary teams, with different levels of contact and support, may generate different effects over time. These findings suggest that future interventions should consider not only duration but also the consistency, personalization, and sustainability of intervention components to maximize long-term benefits. Egger’s regression test suggested the presence of potential publication bias for the HbA1c outcome. However, the pooled effect estimates should be interpreted with caution, the substantial clinical and methodological heterogeneity among the included studies may also have contributed to funnel plot asymmetry.

In addition, several clinical and methodological factors may account for the heterogeneity observed across studies. Cultural modification of the interventions is an important source of heterogeneity. Programs developed in Asia often employed culturally relevant dietary advice, family involvement, or traditional health beliefs (15), whereas studies conducted in Western countries emphasized individualized self-regulation, potentially leading to different effects (86). Therefore, to implement appropriate and effective educational programs, the context of the target population must be considered when developing the program (87). The mode of delivery also substantially varied, ranging from face-to-face education to group sessions and technology-assisted interventions, such as mobile apps, telemonitoring, and text messaging. These differences not only affect accessibility and engagement but may also affect the sustainability of behavior change (88). Previous studies have shown that the combination of in-person intervention and telephone calls can be more effective than applying one mode of delivery alone (89). Likewise, intervention intensity and duration contributed to heterogeneity. Some interventions lasted only four weeks with minimal contact, while others were extended up to two years with frequent follow-up sessions, leading to differences in patients’ adherence and outcomes (90).

In addition to direct blood sugar levels, indicators associated with self-care in T2DM were set as secondary outcomes in this study. The analysis indicated a significant increase in HDL, total cholesterol, BMI, self-care behaviors, and self-efficacy. However, FBG and 2hPG exhibited no significant effect. Consistently, a meta-analysis conducted by Cho et al. revealed that short-term interventions for diabetes can improve health-related outcomes (91). Self-care intervention measures can help improve the treatment of T2DM, maintaining good health and compliance.

Our findings also align with global health priorities. The WHO Global Diabetes Compact (launched in 2021) (92) emphasizes the urgent need for comprehensive diabetes prevention and care, with a focus on equitable access to evidence-based interventions and the integration of self-care into health systems. The efficacy of self-care interventions in reducing HbA1c and improving self-efficacy among working-age adults with T2DM supports these goals, highlighting their role as a sustainable strategy for improving treatment outcomes in resource-constrained settings (93). Furthermore, by explicitly linking theory-based and culturally adapted self-care interventions to global frameworks, our review provides evidence that can guide both national diabetes programs and broader noncommunicable disease (NCD) policies.

### Strengths and limitations

This review has several strengths and limitations. A major strength is the inclusion of RCTs from diverse geographical regions and the application of a theory-informed perspective to evaluate self-management interventions among working-age adults with T2DM. However, several limitations should be acknowledged. Considerable heterogeneity existed across interventions, outcome measures, and follow-up durations, which may influence the interpretation and generalizability of the findings. In addition, variations in intervention components and reporting quality among included studies should be considered when applying these findings to clinical practice.

## Conclusion

This systematic review and meta-analysis indicated that self-care interventions can lead to modest but significant improvements in HbA1c, HDL, total cholesterol, BMI, self-care behavior, and self-efficacy in working-age adults with T2DM. However, substantial heterogeneity, limited blinding, and inconsistent intervention reporting across included trials may affect the interpretation and generalizability of these findings. These findings highlight the need for rigorously designed, theory-informed, and culturally adapted interventions, with transparent reporting and long-term follow-up. Strengthening the methodological quality of future trials is essential to provide more reliable evidence to guide nursing practice, clinical guidelines, and public health strategies for managing T2DM among working-age populations.

## Data Availability

The original contributions presented in the study are included in the article/**Supplementary Material**. Further inquiries can be directed to the corresponding authors.
