# Supplementary material for: The effectiveness of self-care interventions in working-age adults with type 2 diabetes mellitus: a systematic review and meta-analysis
Source: Front Endocrinol (Lausanne). 2026 Sep 11;17:1941060. doi: 10.3389/fendo.2026.1941060 (PMC13612269; doi:10.3389/fendo.2026.1941060)
Supplement: Supplementary file 1 [file SupplementaryFile2.docx]

# Supplementary File 1. Detailed Search Strategy (PubMed Example)

The following search strategy was used in PubMed and adapted for other databases (Embase, CINAHL, Cochrane Library, Web of Science, CBM, CNKI, CQVIP, Wan Fang). The search covered publications from January 2020 to April 2025 and was restricted to English and Chinese language.

## Search terms:

\# 1 ("Diabetes Mellitus"[Mesh]) OR ("Diabetes Mellitus"[Title/Abstract])) OR ("Diabetes Mellitus, Type 2"[Mesh])) OR ("Diabetes Mellitus, Type 2"[Title/Abstract]) OR ("Insulin Resistance"[Mesh]) OR ("Insulin Resistance"[Title/Abstract]) OR (Adult-Onset Diabetes Mellitus[Title/Abstract]) OR (Ketosis-Resistant Diabetes Mellitus[Title/Abstract]) OR (Non-Insulin-Dependent Diabetes Mellitus[Title/Abstract])) OR (Stable Diabetes Mellitus[Title/Abstract]) OR (NIDDM[Title/Abstract])) OR (Maturity Onset Diabetes Mellitus[Title/Abstract])OR (Slow-Onset Diabetes Mellitus[Title/Abstract])

#2 ("Young Adult"[Mesh]) OR ("Young Adult"[Title/Abstract])) OR (young person[Title/Abstract]) OR (young people[Title/Abstract])) OR (early adult[Title/Abstract]))) OR ("Middle Aged"[Mesh]) OR ("Middle Aged"[Title/Abstract]) OR "Working Aged"[Title/Abstract] OR ((18-60 years of age)[Title/Abstract])

#3 ("Self-Management"[Mesh]) OR (self manag*[Title/Abstract])) OR ("self care"[Title/Abstract]) OR ("Self Care"[Mesh])) OR (self caring[Title/Abstract]) OR (self monitor*[Title/Abstract])) OR (self administration[Title/Abstract]) OR (self-medication[Title/Abstract]) OR (self-maintenance[Title/Abstract])) OR ("Self Efficacy"[Mesh]) OR (Self Efficacy[Title/Abstract])

#4 ("Randomized Controlled Trial" [Publication Type]) OR ("Randomized Controlled Trial"[Title/Abstract]) OR ("Controlled Clinical Trial" [Publication Type]) OR ("Controlled Clinical Trial"[Title/Abstract]) OR ("Controlled Clinical Trial"[Title/Abstract]) OR ("Random Allocation"[Mesh]) OR ("Random Allocation"[Title/Abstract]) OR (randomly allocated[Title/Abstract]) OR (RCT[Title/Abstract])

#5 #1 AND #2 AND #3 AND #4

# (CNKI Example)

#1 糖尿病 OR糖尿病, 2型 OR NIDDM OR成熟型糖尿病OR 慢发作型糖尿病OR 2型糖尿病OR 成年型糖尿病OR 非酮症性糖尿病OR 非胰岛素依赖型糖尿病OR 成熟型糖尿病OR  MODY OR稳定性糖尿病

#2 中青年 OR 青年 OR 中年 OR 成年 OR 成人 OR 工作年龄

#3 自我管理 OR 自我护理 OR 自我照护 OR 自我效能 OR 自我调节 OR 自我监测 OR 血糖监测

#4 #1 AND #2 AND #3

Notes:

- Boolean operators (AND, OR) were used to combine concepts.
- Search terms were adapted to database-specific syntax and subject headings.
- Filters applied: 2020–2025, English or Chinese language.
- Reference lists of relevant reviews were hand-searched for additional studies.

# Supplementary File 2. other databases search strategies

## 1. Embase

#1 'diabetes mellitus'/exp OR 'diabetes mellitus':ab,ti OR 'non insulin dependent diabetes mellitus'/exp OR 'non insulin dependent diabetes mellitus':ab,ti OR 'type 2 diabetes':ab,ti OR 't2dm':ab,ti OR 'niddm':ab,ti OR 'maturity onset diabetes mellitus':ab,ti OR 'ketosis resistant diabetes':ab,ti OR 'adult onset diabetes':ab,ti OR 'diabetes mellitus type ii':ab,ti OR 'diabetes mellitus, type 2':ab,ti OR 'insulin independent diabetes':ab,ti OR 'type ii diabetes':ab,ti

#2 'young adult'/exp OR 'young adult' OR 'middle aged'/exp OR 'middle aged':ab,ti OR 'prime adult':ab,ti OR 'young person':ab,ti OR 'university student':ab,ti OR 'college student':ab,ti OR 'working age'/exp OR 'working age':ab,ti OR '18-60 years of age':ab,ti

#3 'self care'/exp OR 'self care' OR 'self monitoring'/exp OR 'self monitor*':ab,ti OR 'self medication'/exp OR 'self manag*':ab,ti OR 'self treatment':ab,ti OR 'self-nurturance':ab,ti OR 'self administration':ab,ti OR 'self-medication':ab,ti OR 'self-maintenance':ab,ti OR 'self concept':ab,ti

#4 'randomized controlled trial'/exp OR 'randomized controlled trial':ab,ti OR 'controlled study'/exp OR 'controlled study':ab,ti OR 'randomization'/exp OR 'randomization':ab,ti OR 'controlled clinical trial':ab,ti OR 'randomly allocated':ab,ti OR rct:ab,ti

#5 #1 AND #2 AND #3 AND #4

## 2. CINAHL (EBSCO host)

S1	(MH "Diabetes Mellitus, Type 2") OR (MH "Diabetes Mellitus") OR (MH "Insulin Resistance") OR SU "Diabetes Mellitus" OR SU "Diabetes Mellitus, Type 2" OR SU "Insulin Resistance" OR SU Adult-Onset Diabetes Mellitus OR SU Ketosis-Resistant Diabetes Mellitus OR SU Ketosis-Resistant Diabetes Mellitus OR SU Stable Diabetes Mellitus OR SU NIDDM OR SU Maturity Onset Diabetes Mellitus OR SU Slow-Onset Diabetes Mellitus

S2	(MH "Young Adult") OR (MH "Middle Age") OR SU "Young Adult" OR SU young person OR SU young people OR SU university student OR SU college student OR SU early adult OR SU "middle aged adults or 45-64 years " OR SU "working aged adults" OR SU 18-60 years of age

S3	(MH "Self-Management") OR (MH "Self Care") OR (MH "Blood Glucose Self-Monitoring") OR (MH "Self Medication") OR SU self-management OR SU self-care OR SU self-regulation OR SU self-monitoring OR SU self-efficacy OR SU self manag* OR SU self monitor* OR SU self-administration OR SU self-medication OR SU self-efficacy

S4	(MH "Randomized Controlled Trials") OR (MH "Clinical Trials") OR (MH "Random Sample") OR (MH "Random Assignment") OR SU Randomized Controlled Trials OR SU "Controlled Clinical Trial" OR SU "Random Allocation" OR SU randomly assigned OR  randomized clinical trial OR SU RCT

S5	S1 AND S2 AND S3 AND S4

## 3. Cochran Library

#1	MeSH descriptor: [Diabetes Mellitus] explode all trees OR MeSH descriptor: [Diabetes Mellitus, Type 2] explode all trees OR ("ketosis resistant diabetes mellitus"):ti,ab,kw OR ("type 2 diabetes mellitus"):ti,ab,kw OR ("non-insulin-dependent diabetes mellitus"):ti,ab,kw OR ("adult-onset diabetes mellitus"):ti,ab,kw OR (Maturity-Onset Diabetes Mellitus):ti,ab,kw OR (Slow-Onset Diabetes Mellitus):ti,ab,kw OR (Stable Diabetes Mellitus):ti,ab,kw OR (Diabetes Mellitus, Type II):ti,ab,kw

#2	MeSH descriptor: [Young Adult] explode all trees OR (young adult):ti,ab,kw OR (young person):ti,ab,kw OR (young people):ti,ab,kw OR ("university student"):ti,ab,kw OR (college student):ti,ab,kw OR MeSH descriptor: [Middle Aged] explode all trees OR (middle aged adults):ti,ab,kw OR (early adult):ti,ab,kw OR (working aged adults):ti,ab,kw

#3	MeSH descriptor: [Self-Management] explode all trees OR MeSH descriptor: [Self Administration] explode all trees OR MeSH descriptor: [Blood Glucose Self-Monitoring] explode all trees OR MeSH descriptor: [Self Efficacy] explode all trees OR(self-management):ti,ab,kw OR ("self care"):ti,ab,kw OR (self-regulation):ti,ab,kw OR (self-monitoring):ti,ab,kw OR (self-efficacy):ti,ab,kw OR (self manag*):ti,ab,kw OR (self monitor*):ti,ab,kw OR (self-administration):ti,ab,kw OR (self-medication):ti,ab,kw

#4	MeSH descriptor: [Randomized Controlled Trial] explode all trees OR MeSH descriptor: [Randomized Controlled Trial] explode all trees OR MeSH descriptor: [Randomized Controlled Trial] explode all trees OR ("randomized controlled trial"):ti,ab,kw OR ("Controlled Clinical Trial"):ti,ab,kw OR ("Random Allocation"):ti,ab,kw OR (randomly allocated):ti,ab,kw OR (RCT):ti,ab,kw OR ("intervention studies"):ti,ab,kw

#5	#1 AND #2 AND #3 AND #4

## 4. Web of science

#1 TS=("Diabetes Mellitus") OR AB=("Diabetes Mellitus") OR TS=("Diabetes Mellitus, Type 2") OR AB=("Diabetes Mellitus, Type 2") OR TS= ("Insulin Resistance") OR AB=("Insulin Resistance") OR AB=(Adult-Onset Diabetes Mellitus) OR AB=(Ketosis-Resistant Diabetes Mellitus) OR AB=(Non-Insulin-Dependent Diabetes Mellitus) OR AB=(Stable Diabetes Mellitus) OR AB=(NIDDM) OR AB=(Maturity Onset Diabetes Mellitus) OR AB=(Slow-Onset Diabetes Mellitus) OR AB=(Type 2 Diabetes Mellitus)

#2 TS=("Young Adult") OR AB=("Young Adult") OR AB=(young person) OR AB=(young people) OR AB=(university student) OR AB=(college student) OR AB=(early adult) OR TS=("Middle Aged") OR AB=("Middle Aged") OR AB=("Working Aged") OR AB=("18-60 years old")

#3 TS=("Self-Management") OR AB=("Self-Management") OR AB=(self manag*) OR AB=("self care") OR TS=("Self Care") OR AB=(self caring) OR AB=(self monitor) OR AB=(self monitor*) OR AB=(self-administration) OR AB=(self-medication) OR AB=(self-maintenance) OR TS= ("Self Efficacy") OR AB=(Self Efficacy)

#4 TS=("Randomized Controlled Trial") OR AB=("Randomized Controlled Trial") OR TS=("Controlled Clinical Trial") OR AB=("Controlled Clinical Trial") OR AB=("Controlled Clinical Trial") OR TS=("Random Allocation") OR AB=("Random Allocation") OR AB=(randomly allocated) OR AB=(RCT)

#5	#1 AND #2 AND #3 AND #4

## 5. CNKI

#1	Diabetes + Mature-type diabetes + Slow-onset diabetes + Type 2 diabetes + Adult-type diabetes + Non-ketotic diabetes + Non-insulin-dependent diabetes + Mature-type diabetes + Stable diabetes

#2	Middle-aged and young people + Middle-aged people + Middle-aged + Young people

#3	Self-management + Self-care + Self-efficacy + Self-regulation + Self-adjustment + Self-control + Self-discipline + Self-monitoring of blood sugar + Self-testing of blood sugar

#4 #1 AND #2 AND #3

## 6. Wan Fang

#1 Diabetes or mature-type diabetes or slow-onset diabetes or type 2 diabetes or adult-type diabetes or non-ketotic diabetes or non-insulin-dependent diabetes or mature-type diabetes or stable diabetes

#2 Young and middle-aged people or middle-aged people or middle age or young people

#3 Self-management or self-care or self-efficacy or self-regulation or self-adjustment or self-control or blood sugar self-monitoring or blood sugar self-testing

#4 #1 AND #2 AND #3

#1 糖尿病 OR糖尿病, 2型 OR NIDDM OR成熟型糖尿病OR 慢发作型糖尿病OR 2型糖尿病OR 成年型糖尿病OR 非酮症性糖尿病OR 非胰岛素依赖型糖尿病OR 成熟型糖尿病OR  MODY OR稳定性糖尿病

#2 中青年 OR 青年 OR 中年 OR 成年 OR 成人 OR 工作年龄

#3 自我管理 OR 自我护理 OR 自我照护 OR 自我效能 OR 自我调节 OR 自我监测 OR 血糖监测

#4 #1 AND #2 AND #3

## 7. CQVIP

#1 Diabetes or mature-type diabetes or slow-onset diabetes or type 2 diabetes or adult-type diabetes or non-ketotic diabetes or non-insulin-dependent diabetes or mature-type diabetes or stable diabetes

#2 Young and middle-aged people or middle-aged people or middle age or young people

#3	Self-management or self-care or self-efficacy or self-regulation or self-adjustment or self-control or blood sugar self-monitoring or blood sugar self-testing

#4	#1 AND #2 AND #3

#1 糖尿病 OR糖尿病, 2型 OR NIDDM OR成熟型糖尿病OR 慢发作型糖尿病OR 2型糖尿病OR 成年型糖尿病OR 非酮症性糖尿病OR 非胰岛素依赖型糖尿病OR 成熟型糖尿病OR  MODY OR稳定性糖尿病

#2 中青年 OR 青年 OR 中年 OR 成年 OR 成人 OR 工作年龄

#3 自我管理 OR 自我护理 OR 自我照护 OR 自我效能 OR 自我调节 OR 自我监测 OR 血糖监测

#4 #1 AND #2 AND #3

## 8. CBM(China Biology Medicine)

#1 Diabetes OR Maturity-onset Diabetes OR Slow-onset Diabetes OR Type 2 Diabetes OR Adult-onset Diabetes OR Non-ketotic Diabetes OR Non-insulin-dependent Diabetes OR Mature-onset Diabetes OR Stable Diabetes

#2 Young and middle-aged people OR middle-aged people OR middle age OR young people

#3 Self-management OR Self-care OR Self-efficacy OR Self-regulation OR Self-adjustment OR Self-control OR Blood glucose self-monitoring OR Blood glucose self-testing

#4 #1 AND #2 AND #3

#1 糖尿病 OR糖尿病, 2型 OR NIDDM OR成熟型糖尿病OR 慢发作型糖尿病OR 2型糖尿病OR 成年型糖尿病OR 非酮症性糖尿病OR 非胰岛素依赖型糖尿病OR 成熟型糖尿病OR  MODY OR稳定性糖尿病

#2 中青年 OR 青年 OR 中年 OR 成年 OR 成人 OR 工作年龄

#3 自我管理 OR 自我护理 OR 自我照护 OR 自我效能 OR 自我调节 OR 自我监测 OR 血糖监测

#4 #1 AND #2 AND #3
